# Molecular Mechanisms Associated with the Induction of Ubiquinone by Peroxisome Proliferators

**DOI:** 10.4021/gr2009.01.1257

**Published:** 2009-01-20

**Authors:** Seher Akhtar Khan

**Affiliations:** Department of Pharmaceutical Sciences, LECOM School of Pharmacy, 1858 West Grandview Blvd., Erie, PA 16509, USA. Email: seherkhan@lecom.edu

## To the editor

Peroxisome proliferators represent a diverse variety of chemicals such as hypolipidemic agents (e.g. clofibric acid, gemfibrozil), phthalate plasticizers, herbicides, adrenal steroids (e.g. dehydroepiandosterone), polyunsaturated fatty acids (and their metabolites), and leukotrienes. These agents, when administered to rats and mice, cause a sustained increase in the size and number of peroxisomes in the liver. In addition, peroxisome proliferators increase liver weight and induce a wide array of genes primarily associated with lipid metabolism, cellular proliferation and defense. High dose feeding of these chemicals for prolonged period can produce liver tumors in the rodents. The biochemical and cellular effects of these chemicals, however, are only observed in mice and rats, and not in most other animals and man.

Peroxisome proliferators elicit their pleiotropic effects through activation of peroxisome proliferator activated-receptor alpha (PPARα), a transcription factor expressed abundantly in the hepatic tissues. Upon activation, PPARα forms a heterodimer with retinoid X receptor alpha (RXRα), and regulates numerous genes by binding to PPAR-response elements (PPRE), located upstream on the responsive genes [1]. Hepatic responses to peroxisome proliferating chemicals (i.e. peroxisome proliferation, enzyme induction) can be completely abolished in an experimental mouse model where functional PPARα is disrupted [2].

Previous studies have reported that livers of rats and mice treated with peroxisome proliferators contain a substantially high concentration of ubiquinone, as compared to control animals [3]. Ubiquinone, a mevalonate-derived polyisoprenoid, is an essential component of mammalian cells. This lipid molecule consists of repeats of isoprenoid unit linked to a quinone ring. Ubiquinone-9, which contains a side chain of 9 repeats of isoprene residues, is the major form in rat tissues, whereas ubiquinone-10 (with 10 isoprene subunits) predominates in the humans. The major site of ubiquinone biosynthesis is the endoplasmic reticulum-Golgi system, although biosynthetic enzymes have also been detected in the peroxisomes and mitochondria.

The biological role of ubiquinone in the mitochondrial electron transport system has been extensively studied and well documented. In addition, it serves as a potent cellular antioxidant and maintains the integrity of biological membranes.

Ubiquinone exerts antioxidative effects by a combination of mechanisms one of which is by inhibition of lipid peroxidation in biological membranes. Ubiquinone inhibits initiation and propagation of lipid peroxidation by donating reducing equivalents. Additionally, ubiquinone is involved in the recycling of vitamin E from tocopheroxyl radical and facilitates cellular uptake of vitamin E.

By virtue of these properties, ubiquinone can protect the cellular environment from oxidative damage. Also, since the cellular uptake of this lipid from diet is minimal, there is a growing interest to increase its endogenous production.

While attempting to elucidate the mechanism of peroxisome proliferator-mediated increased level of hepatic ubiquinone, genetically engineered knockout mouse models for nuclear receptors PPARα and RXRα have been utilized [4, 5]. A marked increase in hepatic ubiquinone was observed in wild-type mice exposed to a peroxisome proliferator, diethylhexylphthalate (DEHP). In contrast, DEHP treatment did not alter its concentration in the liver of PPARα-knockout mice [4]. Tracer studies with [^3^H] mevalonate precursor indicated that the increase of ubiquinone by DEHP was a result of increased biosynthesis. In DEHP-treated mice, the incorporation of radio-tag in the liver was three times higher than wild-type control mice. Similarly, the activities of some branch point enzymes associated with mevalonate pathway were markedly increased only in the DEHP-treated wild-type mice. It is interesting to note that the basal level of ubiquinone was unaffected by ablation of the nuclear hormone receptor [4]. Although the receptor protein is not critical for basal biosynthesis of ubiquinone, it is critical in ubiquinone induction following exposure to peroxisome proliferators and possibly other chemicals.

A striking contrast was observed with liver-specific RXR-knockout mouse model. In this model, ubiquinone concentration was decreased by half as compared to control wild-type mice, which resulted from a lower basal level of biosynthesis [5]. This data supports the role of RXRα in physiological regulation of ubiquinone synthesis. Following treatment with DEHP, ubiquinone concentration was markedly elevated in the livers of both control and RXRα- knockout mice. It may be possible that DEHP is metabolized to a PPARα activator or may induce an endogenous PPARα ligand, resulting in a significant increase of hepatic ubiquinone in RXRα-null mice. Whether other peroxisome proliferators elicit similar responses as those of DEHP remains to be determined.

Maintenance of basal transcription of ubiquinone in the absence of PPARα may result from recruitment of other receptors and cofactors. In fact, RXRα can serve as dimerization partner of other nuclear receptors e,g, liver X receptor (LXR), farnesoid X receptor (FXR) and thyroid hormone receptor. Future studies are therefore necessary to understand the precise mechanism of the physiological regulation of ubiquinone.

In summary, functional PPARα is an absolute requirement to increase the hepatic levels of ubiquinone by peroxisome proliferators. The receptor serves as a necessary cellular element for pleiotropic effects of these chemicals. RXRα, on the other hand is associated with homeostatic regulation of ubiquinone.
